# Predicting exacerbation of renal function by DNA methylation clock and DNA damage of urinary shedding cells: a pilot study

**DOI:** 10.1038/s41598-024-62405-4

**Published:** 2024-05-21

**Authors:** Akihito Hishikawa, Erina Sugita Nishimura, Norifumi Yoshimoto, Ran Nakamichi, Eriko Yoshida Hama, Wataru Ito, Tomomi Maruki, Kengo Nagashima, Ryoko Shimizu-Hirota, Hiromasa Takaishi, Hiroshi Itoh, Kaori Hayashi

**Affiliations:** 1 Division of Nephrology, Endocrinology and Metabolism, Department of Internal Medicine, Keio University School of Medicine, 35 Shinanomachi, Shinjuku-Ku, Tokyo, 160-8582 Japan; 2 Biostatistics Unit, Clinical and Translational Research Center, Keio University School of Medicine, Tokyo, Japan; 3 Center for Preventive Medicine, Keio University School of Medicine, Tokyo, Japan

**Keywords:** Nephrology, Biomarkers

## Abstract

Recent reports have shown the feasibility of measuring biological age from DNA methylation levels in blood cells from specific regions identified by machine learning, collectively known as the epigenetic clock or DNA methylation clock. While extensive research has explored the association of the DNA methylation clock with cardiovascular diseases, cancer, and Alzheimer's disease, its relationship with kidney diseases remains largely unexplored. In particular, it is unclear whether the DNA methylation clock could serve as a predictor of worsening kidney function. In this pilot study involving 20 subjects, we investigated the association between the DNA methylation clock and subsequent deterioration of renal function. Additionally, we noninvasively evaluated DNA damage in urinary shedding cells using a previously reported method to examine the correlation with the DNA methylation clock and worsening kidney function. Our findings revealed that patients with an accelerated DNA methylation clock exhibited increased DNA damage in urinary shedding cells, along with a higher rate of eGFR decline. Moreover, in cases of advanced CKD (G4-5), the DNA damage in urinary shedding cells was significantly increased, highlighting the interplay between elevated DNA damage and eGFR decline. This study suggests the potential role of the DNA methylation clock and urinary DNA damage as predictive markers for the progression of chronic kidney disease.

## Introduction

Chronic kidney disease (CKD) is becoming a significant global health concern, driven by an aging population and the increasing prevalence of hypertension and diabetes^1^. Although renal function can be evaluated using serum creatinine and cystatin C, the current markers for predicting future deterioration of kidney function are limited to urine protein levels and pathological findings from kidney biopsies. Therefore, there is a pressing need to identify novel markers to predict CKD progression.

Epigenetic changes, including DNA methylation, have been implicated in the pathogenesis of hypertension and renal disease^2^. Our previous research has focused on the pathophysiological significance of DNA damage and DNA methylation changes in kidney organizing cells. We were the first to discover that the DNA repair factor lysine acetyltransferase 5 (KAT5) is essential for maintaining podocyte integrity, and its decreased expression in diabetic nephropathy leads to DNA damage and DNA methylation changes^3^. Additionally, we reported that KAT5 plays a crucial role in proximal tubular cells, contributing to the prevention of acute kidney injury (AKI) through the epigenetic regulation of K-Cl cotransporter 3 (KCC3)^4^. In addition to these basic medical studies, we conducted studies using human clinical samples. We reported associations between DNA double-strand breaks (DSBs) and DNA methylation levels of glomeruli with 1-year eGFR decline in IgA nephropathy patients^5^. We also explored the significance of DSBs in membranous nephropathy and diabetic nephropathy^6^. Furthermore, we reported a method for noninvasive detection of DNA double-strand breaks (DSBs) in kidney organizing cells using urinary shedding cells^7^.

More recently, we discovered that podocyte-specific induction of DNA double-strand breaks (DSBs) triggers DNA methylation changes in blood cells, contributing to further exacerbation of renal pathologies^8^. While several human epigenome-wide association studies (EWASs) have reported a potential association between DNA methylation changes in blood cells and kidney function^9–11^, the underlying mechanisms have remained elusive. Our findings revealed that DNA damage in kidney cells may induce DNA methylation changes in blood cells.

Recent reports have suggested the possibility of estimating biological age from the DNA methylation profiles of various tissues, including blood cells, known as the epigenetic clock or DNA methylation clock^12,13^. The epigenetic clock, determined by machine learning in specific DNA methylation regions, serves as an indicator of biological aging, with the difference between the epigenetic clock and chronological age serving as a biomarker for biological aging^14^. Although associations with cardiovascular diseases^15^, Alzheimer's disease^16^, and cancers^17^ have been reported, the significance of the epigenetic clock in kidney diseases remains unclear. In addition, another group reported that mice transiently expressing I-PpoI, an enzyme that induces DNA damage, show an accelerated epigenetic clock and aging phenotype^18^.

Considering the recent attention to the relationship between DNA damage and the epigenetic clock, we aimed to investigate DNA methylation age and urinary shedding cell DNA damage as predictors of worsening kidney function.

## Results

### Participant characteristics

Individuals who visited the Center for Preventive Medicine for annual health checkups (healthy controls) or the outpatient clinic of the Department of Nephrology at Keio University Hospital were enrolled. A total of 20 participants (non-CKD 8, CKD 12) aged 62.9 ± 2.0 years old were eligible for this study. Table 1 shows the general characteristics of the subjects. The eGFR of the control group was 70.4 ± 5.9 (ml/min/1.73 m^2^), which was slightly lower, but did not contradict previous reports as a decline in renal function associated with aging^19^. There was no significant difference in chronological age between healthy subjects and CKD patients, and there was no correlation between eGFR and chronological age or DNA methylation age (Supplementary Fig. 1A–C). Patients with a history of malignant disease, Parkinson's disease, or Alzheimer's disease, which are reported to be associated with epigenetic clock progression, were excluded from the study.

**Table 1:** Participant characteristics.

	Non-CKD	CKD	p value
N	8	12	
Age	62.6 ± 3.2	63.1 ± 2.6	0.91
Hannum’s clock	57.9 ± 2.3	61.1 ± 1.9	0.28
PhenoAge	52.7 ± 3.3	54.5 ± 2.7	0.67
Sex (male) (%)	5 (62.5%)	12 (100%)	0.049*
BMI (kg/m^2^)	23.4 ± 1.0	23.2 ± 0.8	0.88
Systolic BP (mmHg)	121.3 ± 8.5	137.3 ± 7.0	0.16
Diastolic BP (mmHg)	74.4 ± 3.6	79.6 ± 2.9	0.27
eGFR (ml/min/1.73 m^2^)	70.4 ± 5.9	32.2 ± 4.8	< 0.0001
eGFR slope (ml/min/1.73 m^2^/year)	0.5 ± 1.2	− 2.1 ± 1.0	0.11
Albumin-to-creatinine ratio (ACR) (mg/g Cr)	4.5 ± 243.5	977.2 ± 198.8	0.0063*
Diabetes (%)	1 (12.5%)	2 (16.7%)	0.66
Hypertension (%)	2 (25%)	7 (58.3%)	0.16
Dyslipidemia (%)	5 (62.5%)	3 (25%)	0.11
CKD stage		G3a: 4 (33.3%)	
		G3b: 2 (16.7%)	
		G4: 3 (25.0%)	
		G5: 3 (25.0%)	
Cause of CKD		Hypertensive nephropathy: 5 (41.6%)	
		Diabetic nephropathy: 2 (16.7%)	
		Chronic glomerulopathy: 3 (25%)	
		Indeterminate: 2 (16.7%)	

*p < 0.05.

### Correlation between chronological age and epigenetic clocks

**Figure 1:**
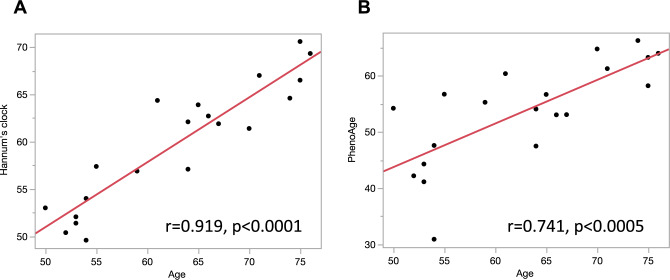
Correlation between chronological age and epigenetic clocks. Univariate logistic regression analysis showing the association between chronological age and epigenetic clocks. (**A**) Hannum’s clock. (**B**) PhenoAge.

DNA was extracted from 2 ml of patient blood as described in the “Methods” section, and DNA methylation changes were evaluated with the EPIC array. We calculated DNA methylation clocks from the DNA methylation information in the EPIC array, focusing on specific CpGs according to a previously reported method. In this study, we analyzed Hannum's clock^12^, which is the first-generation DNA methylation clock and has been used in various aging studies. Additionally, we examined PhenoAge^13^, a second-generation DNA methylation clock developed by Levine M. The results of the analysis showed that both Hannum's clock and PhenoAge showed a strong correlation with chronological age (Hannum's clock: r = 0.919, p < 0.0001, PhenoAge: r = 0.741, p < 0.0005) consistent with previous reports (Fig. 1A,B).

### Relationship between DNA DSBs of urinary shedding cells and renal function or eGFR slope

**Figure 2:**
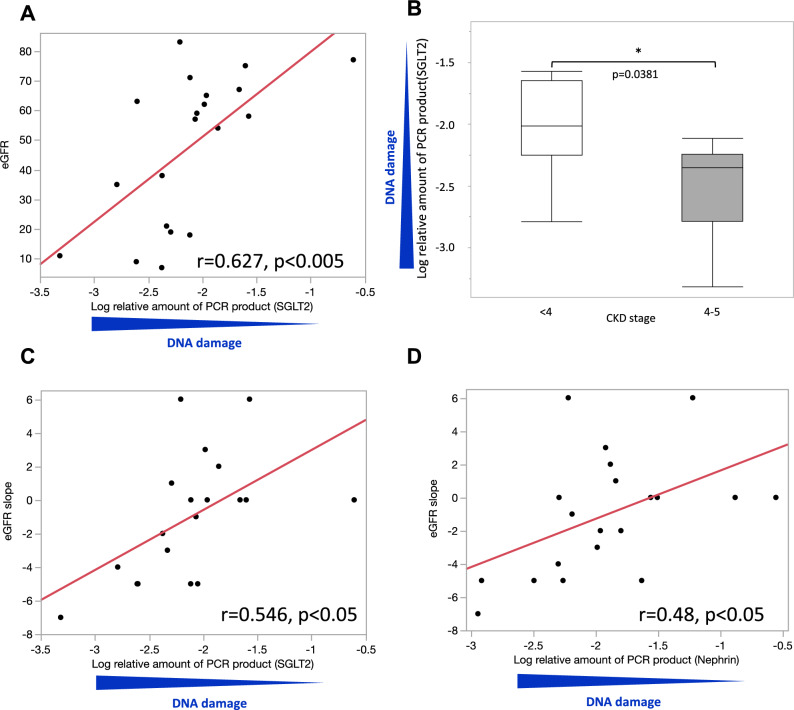
Relationship between DNA DSBs of urinary shedding cells and renal function or eGFR slope. (**A**) Univariate logistic regression analysis showing the association between DNA DSBs of the SGLT2 gene and eGFR. (**B**) Detectable increase in DNA DSB sites in the SGLT2 gene evaluated by the long-distance PCR method in patients with CKD G4-5. (**C**) Univariate logistic regression analysis showing the association between DNA DSBs of the SGLT2 gene and eGFR slope. (**D**) Univariate logistic regression analysis between DNA DSBs of the nephrin gene and eGFR slope.

DNA DSBs of urinary shedding cells were evaluated by the quantitative long-distance PCR method as described in the “Methods” section. Briefly, this method for detecting DNA DSBs is based on the assumption that DNA with fewer DSB lesions will amplify to a greater extent than more damaged DNA if equal amounts of DNA from different samples are amplified under identical conditions^7,20^. A significant negative correlation was observed between DNA DSBs in urinary shedding cells, especially in the proximal tubules, and eGFR (r = 0.627, p < 0.005) (Fig. 2A). Furthermore, in advanced CKD G4 or higher, there was a significant increase in DNA DSBs in urinary proximal tubular cells (Fig. 2B). These trends were more evident in the proximal tubules than in DNA DSBs in urinary podocytes, suggesting the physiological importance of DNA damage in the proximal tubules (Supplementary Fig. 2A,B). We also examined the association between DNA damage in urinary shedding cells and the eGFR slope. The eGFR slope was calculated from all available eGFR measurements after the measurement of DNA methylation age for each participant using linear regression, with requirement of a minimum of 2 eGFR measurements separated by at least 1 year^21,22^. The results showed that increased DNA DSBs in urinary proximal tubular cells and podocytes were significantly correlated with the eGFR slope (DNA damage of SGLT2 gene: r = 0.546, p < 0.05, DNA damage of nephrin gene: r = 0.48, p < 0.05) (Fig. 2C,D).

### Relationship between age acceleration and DNA DSBs of urinary shedding cells and eGFR slope

**Figure 3:**
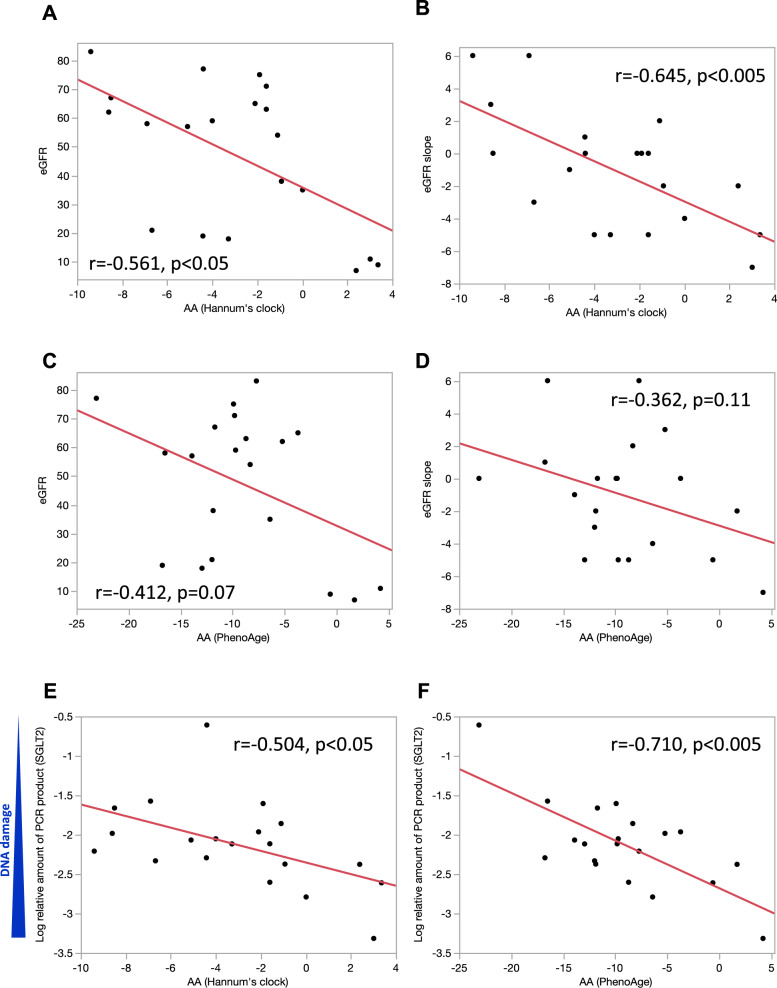
Relationship between age acceleration and DNA DSBs of urinary shedding cells or eGFR slope. (**A**, **B**) Univariate logistic regression analysis showing the association between age acceleration (AA) measured by Hannum’s clock and eGFR or eGFR slope. (**C**, **D**) Univariate logistic regression analysis showing the association between age acceleration (AA) measured by PhenoAge and eGFR or eGFR slope. (**E**, **F**) Univariate logistic regression analysis showing the association between age acceleration (AA) and DNA DSBs of the SGLT2 gene.

**Figure 4:**
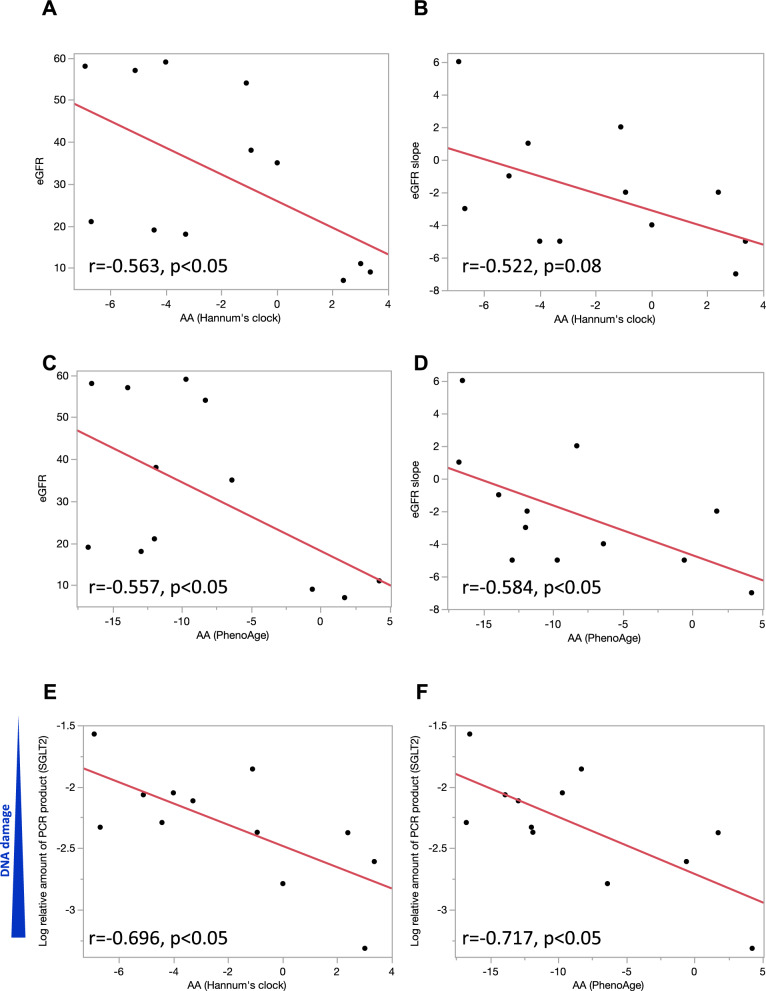
Relationship between age acceleration and DNA DSBs of urinary shedding cells or eGFR slope in CKD patients. (**A**, **B**) Univariate logistic regression analysis showing the association between age acceleration (AA) measured by Hannum’s clock and eGFR or eGFR slope. (**C**, **D**) Univariate logistic regression analysis showing the association between age acceleration (AA) measured by PhenoAge and eGFR or eGFR slope. (**E**, **F**) Univariate logistic regression analysis showing the association between age acceleration (AA) and DNA DSBs of the SGLT2 gene.

The difference between DNA methylation age and chronological age, termed age acceleration (AA), is known to be a strong predictor of lifespan and healthspan^23,24^. We evaluated the relationship between AA and DNA damage of urinary shedding cells, eGFR and eGFR slope. AA measured by Hannum’s clock negatively correlated with eGFR (r = − 0.561, p < 0.05) and eGFR slope (r = − 0.645, p < 0.005) (Fig. 3A,B). AA measured by PhenoAge also showed tendency towards negative correlation with eGFR (r = − 0.412, p = 0.07) and eGFR slope (r = − 0.362, p = 0.11) (Fig. 3C,D). Moreover, it also showed a significant correlation with DNA damage in urinary shedding cells (correlation between AA measured by Hannum’s clock and DNA damage of SGLT2 gene: r = − 0.504, p < 0.05, correlation between AA measured by PhenoAge and DNA damage of SGLT2 gene: r = − 0.710, p < 0.005) (Fig. 3E,F, Supplementary Fig. 3A,B). Similar associations were observed when the analysis was confined to the CKD group alone, yielding results consistent with those presented in Fig. 3 (Fig. 4A–F). These results indicate that increased DNA damage in urinary shedding cells and accelerated biological aging calculated from the DNA methylation clock may be indicators of current renal function and predictive markers of future renal function exacerbations.

## Discussion

In this study, we evaluated the DNA methylation clock and DNA DSBs in urinary shedding cells using blood and urine samples from healthy subjects and CKD patients. The results indicated that accelerated biological aging calculated from DNA methylation clocks and increased DNA DSBs of urinary shedding cells may serve as markers for current renal function and the prediction of future renal function deterioration. We also found that DNA DSBs in urinary shedding cells were associated with accelerated DNA methylation age.

The DNA methylation clock is a biological age calculated from DNA methylation levels, focusing on 71 specific CpGs in Hannum's clock^12^ and 391 in PhenoAge^13^. PhenoAge is a second-generation clock that has been further trained for aging phenotypes and is superior to the first-generation clock in predicting many aging-related diseases and lifespan. In addition, age acceleration (AA), the difference between the DNA methylation clock and chronological age, has been used in many studies as an indicator of biological aging^23,24^.

To evaluate DNA DSBs in the kidney, we extracted genomic DNA from urinary shedding cells and evaluated it by a quantitative long-distance PCR method that we previously reported^7^. Open chromatin regions of active transcription, such as promoter regions of cell-specific marker genes, are known to be vulnerable to DNA damage. Therefore, we used primer sets of the promoter region of sodium glucose cotransporter 2 (SGLT2) and nephrin, which are expressed specifically in proximal tubular cells and podocytes, to assess DNA DSBs in each cell.

The analysis revealed a negative correlation between DNA DSBs of the urinary proximal tubular cells and eGFR and that DNA DSBs were significantly increased in advanced CKD. Interestingly, DNA damage in the proximal tubules was more associated with eGFR than that in podocytes. This may be related to the fact that proximal tubular cells are energy-consuming cells that reabsorb solutes, and in general, energy-consuming cells are more likely to accumulate oxidative damage, which is known to be a cause of age-related diseases. As CKD advances, tubulointerstitial fibrosis becomes a defining feature, a characteristic shared by all forms of CKD and renal fibrosis. Importantly, the severity of tubulointerstitial damage has been demonstrated to have a stronger correlation with the reduction in GFR compared to the extent of glomerular injury^25^.

Furthermore, the extent of DNA damage in urinary proximal tubular cells and podocytes exhibited a negative correlation with eGFR slope. Additionally, the degree of biological aging assessed through age acceleration (AA) also displayed a negative correlation with eGFR and eGFR slope. Moreover, there was a significant correlation between the extent of DNA damage in urinary shedding cells and age acceleration (AA). These findings suggest that the escalation of DNA damage in urinary shedding cells and the acceleration of biological aging calculated from the DNA methylation clock could potentially serve as indicators for current renal function and the prediction of future renal function deterioration. In particular, the DNA methylation clock acceleration demonstrated a strong correlation with eGFR slope and current eGFR, suggesting its potential as a predictive marker for future renal function deterioration. To our knowledge, this is the first report demonstrating the potential of DNA methylation clock progression as a marker for predicting future renal function decline and its association with DNA damage in urinary shedding cells.

Although the association between progression of the DNA methylation clock and increased mortality rate^23,24^, cancer^17^, and Alzheimer's disease^16^ has been previously reported, the relationship with renal function has not been well studied. Recent studies have reported accelerated aging in CKD and that inflammatory and immunologic profiles may be important in predicting biological aging^26^. In addition, a large meta-analysis in Europeans, African Americans, and Hispanics reported an association between the progression of DNA methylation age and eGFR or CKD^27^. The results of our study targeting an Asian population are consistent with the findings previously reported. It is widely acknowledged that kidney function declines with age, and some kidney diseases are known to have a poor renal prognosis with aging^28^. The correlation between biological aging indicated by the DNA methylation clock and the decrease in eGFR is a reasonable finding. The present study provides new findings that biological aging is associated not only with eGFR but also with eGFR slope, which may be a marker for predicting future renal function. Moreover, recent reports have proposed the possibility that chronic kidney disease promotes aging in a multiorgan disease network^29^. Notably, this is the first study to demonstrate a link between DNA damage in kidney cells and biological aging. Although this study cannot demonstrate a causal relationship between kidney DNA damage and the DNA methylation clock and systemic aging, it has been reported that DNA damage in podocytes induces DNA methylation changes in blood cells^8^ and that the DNA methylation clock progresses when systemic DNA damage is induced^18^, suggesting that DNA damage in kidney cells may be related to systemic aging via peripheral blood DNA methylation changes.

Recent studies have suggested that DNA damage in kidney cells contributes to the progression of renal fibrosis and kidney dysfunction. For instance, Kishi et al. demonstrated that in a mouse model, deletion of ATR, a key regulator of the DNA damage response, specifically in renal proximal tubular epithelial cells (RPTECs) led to increased DNA damage, apoptosis, acute kidney injury, and fibrosis following renal insults such as cisplatin treatment, ischemia–reperfusion injury, and unilateral ureteral obstruction^30^. They also found that ATR expression inversely correlated with DNA damage in kidney tissues from patients with chronic kidney disease. Furthermore, Gupta et al. recently reported using human kidney organoids that FANCD2, a DNA repair factor involved in homologous recombination, was decreased in tubular cells in the setting of incomplete repair, correlating with the degree of fibrosis^31^.

Moreover, in our current study, we demonstrated that podocyte-specific deletion of ERCC1, a key factor for DNA single-strand break repair, in mice resulted in severe proteinuria, glomerulosclerosis, and renal failure, accompanied by accumulation of DNA damage^32^. Restoration of podocyte ERCC1 expression attenuated proteinuria and glomerulosclerosis with reduced DNA damage.

Collectively, these findings highlight the potential of targeting DNA damage pathways, such as via restoring expression of repair factors like KAT5, ATR, FANCD2 and ERCC1 in kidney cells, as a therapeutic approach to slow down the progression of kidney disease in both tubular cells and podocytes.

However, this study has several limitations. In particular, the sample size was small, and long-term renal prognosis was not followed up. Further studies are needed to evaluate the association of DNA DSBs in urinary shedding cells and the DNA methylation clock with eGFR and worsening renal function, which would require a longer observation period in a larger population. Despite these limitations, this study holds significance in demonstrating the correlation between the acceleration of biological aging evaluated by the DNA methylation clock and the increase in DNA damage in kidney cells. It also indicates the possibility that these factors could serve as indicators for current kidney function assessment and as predictive markers for future renal function deterioration. Furthermore, although a causal relationship could not be established, the findings suggest that kidney DNA damage and renal dysfunction might be implicated in the progression of the DNA methylation clock, contributing to systemic aging. The noninvasive detection of kidney DNA damage allows for the possibility of not only predicting kidney outcomes but also predicting systemic aging progression.

## Methods

### Study population

Individuals aged 50–76 years old who visited the Center for Preventive Medicine for annual health checkups (healthy controls) or the outpatient clinic of the Department of Nephrology at Keio University Hospital were enrolled. Patients with CKD were included in this study. We excluded participants without essential data, including age, sex, body mass index (BMI), systolic blood pressure (BP), diastolic BP and serum chemistry profiles. Individuals with malignant disease, Parkinson's disease and Alzheimer's disease were also excluded. In total, data from 20 participants (non-CKD 8, CKD 12) were included and analyzed.

### Clinical evaluation and laboratory measurements

Blood pressure was measured on the right upper arm after subjects had rested for at least 5 min in a sitting position in the hospital. Blood pressure was measured with an automatic device (BP-900) with the combination of the Korotkoff sounds method and oscillometric technique (TANITA Co. Tokyo, Japan). Blood and urine samples were collected at the same visit. The samples were collected and immediately analyzed using standard hospital laboratory techniques at Keio University Hospital.

### Definitions

eGFR was calculated using the following equation: eGFR (ml/min/1.73 m^2^) = 194 × serum creatinine (− 1.094) × age (− 0.287) × 0.739 (if female). Hypertension was defined as systolic BP ≥ 140 mmHg and/or diastolic BP ≥ 90 mmHg or the use of antihypertensive drugs. Diabetes was defined in accordance with the guidelines of the American Diabetes Association as a fasting glucose concentration ≥ 126 mg/dl, HbA1c level ≥ 6.5% or the use of antihyperglycemic drugs. The eGFR slope was calculated from the eGFR one year after the blood sample was collected for calculation of the DNA methylation clock.

### Urine sample collection

Fifty milliliters of urine samples were prospectively collected from the outpatients who signed a consent form at the clinic visit. Urine samples were collected from each participant and processed immediately without freezing to maintain the integrity of the cellular components and prevent crystal formation. The samples used were left over from routine urine collections, which were midstream samples. All samples were assigned a study number that connected them to clinical information from the chart to deidentify them.

### Urine processing

Urine was centrifuged at 4 °C for 15 min at 3,500 rpm. The urine pellet was suspended in 500 μl of cold diethylpyrocarbonate-treated PBS (pH 7.4) at 4 °C. A second 500 μl aliquot of PBS was added to wash the bottom of the 50 mL centrifuge tube to recover the remaining pellet material. The transferred pellet material in 1.0 ml of PBS was centrifuged at 12,000 rpm for 5 min at 4 °C, and the pellet was used for DNA analysis. DNA was extracted with the NucleoSpin Tissue kit (Takara, Japan) according to the manufacturer’s instructions.

### Long-distance PCR

The quantitative long-distance PCR method for detecting DNA DSBs is based on the assumption that DNA with fewer DSB lesions will amplify to a greater extent than more damaged DNA if equal amounts of DNA from different samples are amplified under identical conditions, as described previously. We created primers to amplify an approximately 10,000-bp section of the promoter region of the cell-specific marker genes, as described in Supplementary Table 1. Real-time RT-PCR was performed using SYBR Green PCR Master Mix (Applied Biosystems) and run using StepOne devices from Applied Biosystems (Thermo Fisher Scientific). The amount of PCR product was evaluated by the standard curve and adjusted by the level of the GAPDH gene, which effectively minimizes the influence of variations in sample concentration and DNA template amount.

### DNA extraction, quantification, and methylation assessment

Genomic DNA was extracted from whole blood cells using the QIAamp DNA Blood Mini 139 Kit (Qiagen, USA). DNA methylation was assayed on the Illumina Infinium MethylationEPIC array. The standard manufacturer’s protocol was used and carried out by Takara Bio Inc. (Japan).

### DNA methylation age

We estimated two different types of methylation age using Hannum’s DNAm age (Hannum et al., 2013) and PhenoAge (Levine et al., 2018). We used R software (version 4.1.3; R Foundation for Statistical Computing, Vienna, Austria) with the packages ‘BiocManager (version 1.30.16)’ and ‘ENmix (version 1.30.03)’ to calculate the epigenetic aging clocks.

### Statistical analysis

For univariate analyses, analysis of variance (ANOVA) followed by Scheffe’s post hoc test and Pearson’s test were used for categorical and continuous variables, respectively. Continuous variables are expressed as the mean ± standard error of the mean (SEM). All statistical analyses were performed using JMP version 14 (SAS Institute Inc., Cary, NC, USA).

### Study approval

The Ethics Committee of Keio University School of Medicine approved the present study (approval number: 2020-0147). Written informed consent was obtained from participants prior to inclusion in the study. Participants were identified using a number rather than their name. This study was conducted in adherence with the Declaration of Helsinki. All methods were carried out in accordance with the institutional guidelines of the ethics committee at Keio University (Tokyo, Japan).

## Supplementary Information

Supplementary Figures.

Supplementary Table S1.

## Data Availability

The datasets used and/or analysed during the current study are available from the corresponding author on reasonable request.
