# Author Correction: Predicting exacerbation of renal function by DNA methylation clock and DNA damage of urinary shedding cells: a pilot study

**DOI:** 10.1038/s41598-024-84241-2

**Published:** 2024-12-31

**Authors:** Akihito Hishikawa, Erina Sugita Nishimura, Norifumi Yoshimoto, Ran Nakamichi, Eriko Yoshida Hama, Wataru Ito, Tomomi Maruki, Kengo Nagashima, Ryoko Shimizu‑Hirota, Hiromasa Takaishi, Hiroshi Itoh, Kaori Hayashi

**Affiliations:** 1 Division of Nephrology, Endocrinology and Metabolism, Department of Internal Medicine, Keio University School of Medicine, 35 Shinanomachi, ShinjukuKu, Tokyo 1608582 Japan; 2 Biostatistics Unit, Clinical and Translational Research Center, Keio University School of Medicine, Tokyo, Japan; 3 Center for Preventive Medicine, Keio University School of Medicine, Tokyo, Japan

Correction to: *Scientific Reports* 10.1038/s41598-024-62405-4, published online 21 May 2024

The Acknowledgements section in the original version of this Article was incomplete.

“We acknowledge Dr. Hideaki Nakaya for assistance during the initial stage of this study. This study was supported by the Grants for Scientific Research (22H03091) from the Ministry of Education, Culture, Sports, Science and Technology (MEXT) of Japan and a grant from the Japan Foundation for Applied Enzymology.”

now reads:

"We acknowledge Dr. Hideaki Nakaya for assistance during the initial stage of this study. This study was supported by the Grants for Scientific Research (22H03091) from the Ministry of Education, Culture, Sports, Science and Technology (MEXT) of Japan, the JST FOREST Program (Grant number JPMJFR210V, Japan) and a grant from the Japan Foundation for Applied Enzymology.”

The original Article has been corrected.

