# Supplementary material for: Predicting exacerbation of renal function by DNA methylation clock and DNA damage of urinary shedding cells: a pilot study
Source: Sci Rep. 2024 May 21;14:11530. doi: 10.1038/s41598-024-62405-4 (PMC11109093; doi:10.1038/s41598-024-62405-4)
Supplement: Supplementary file 1 — Supplementary Figures. [file 41598_2024_62405_MOESM1_ESM.pdf]

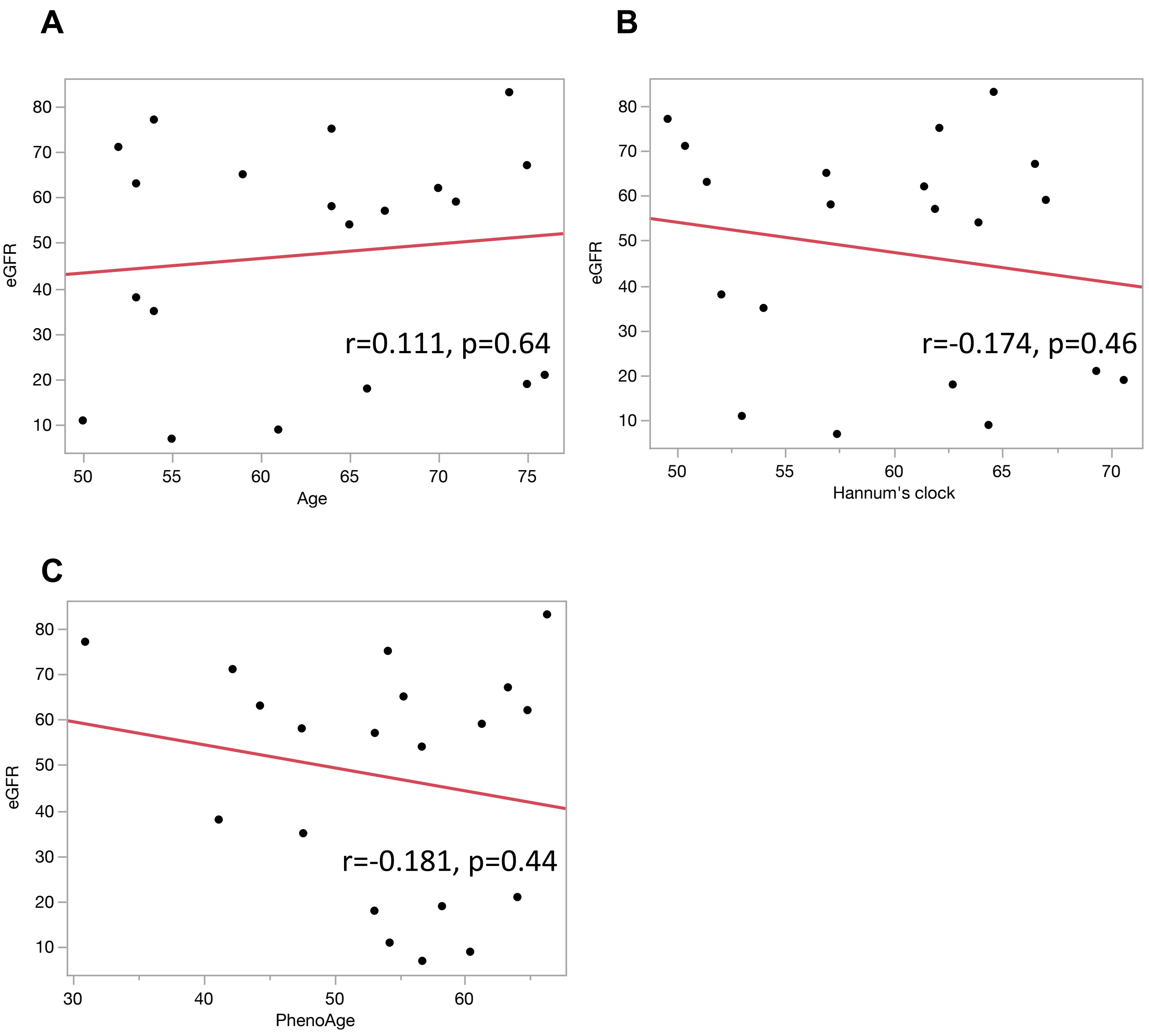

Supplementary Figure 1. Correlation between chronological age or epigenetic clocks and eGFR.

(A) Chronological age. (B) Hannum’s clock. (C) PhenoAge.

Suppl Fig. 2

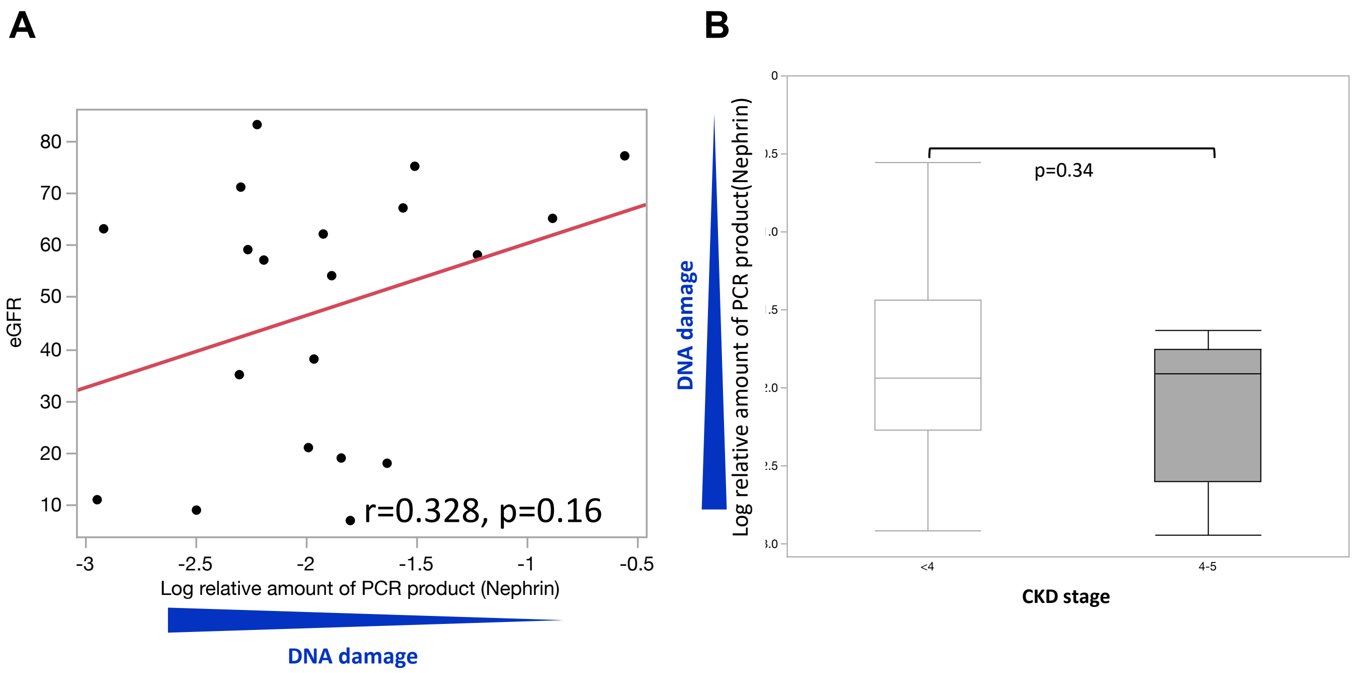

Supplementary Figure 2. Relationship between DNA DSBs of urinary shedding cells and renal function or eGFR slope.

(A) Univariate logistic regression analysis between DNA DSBs of the nephrin gene and eGFR. (B) DNA DSB sites in the nephrin gene evaluated by the long-distance PCR method in patients with CKD G4-5.

Suppl Fig. 3

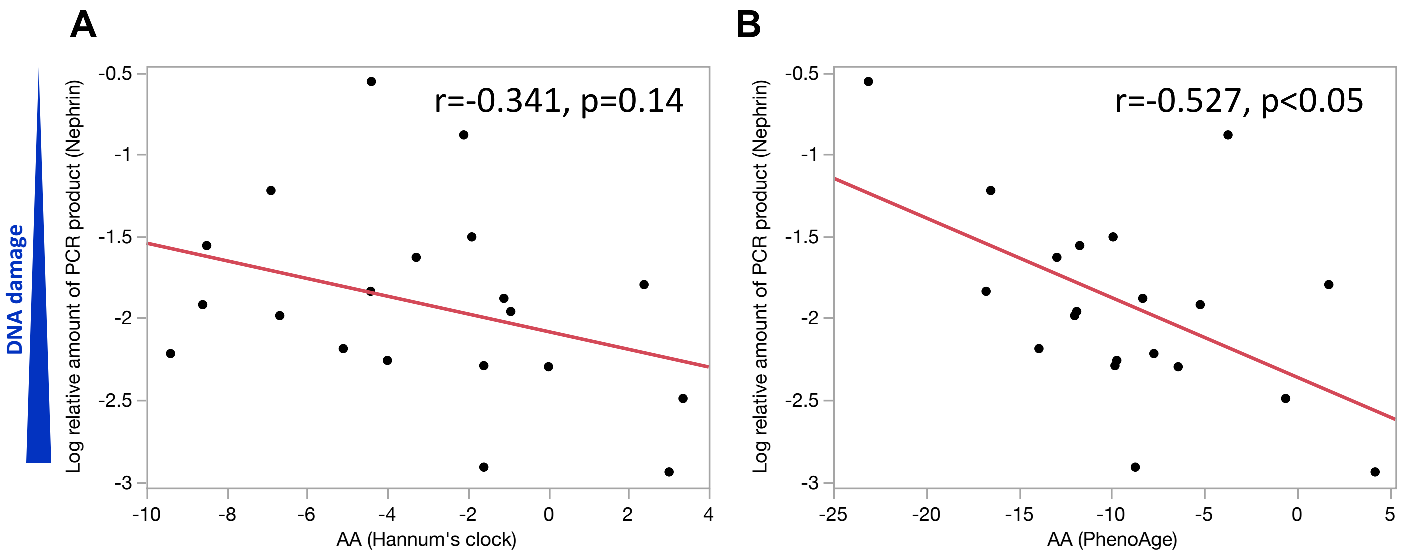

Supplementary Figure 3. Relationship between age acceleration and DNA DSBs of urinary shedding cells.

Univariate logistic regression analysis showing the association between age acceleration (AA) and DNA DSBs of the nephrin gene. (A) AA measured by Hannum's clock. (B) AA measured by PhenoAge.
