# Supplementary material for: Predicting exacerbation of renal function by DNA methylation clock and DNA damage of urinary shedding cells: a pilot study
Source: Sci Rep. 2024 May 21;14:11530. doi: 10.1038/s41598-024-62405-4 (PMC11109093; doi:10.1038/s41598-024-62405-4)
Supplement: Supplementary file 2 — Supplementary Table S1. [file 41598_2024_62405_MOESM2_ESM.docx]

**SUPPLEMENTARY TABLES**

**Supplementary Table 1. Gene-specific primer pairs used for quantitative long-distance PCR**

| **Name** | **F/R** | **Sequence** |
| --- | --- | --- |
| GAPDH | F | GCCCTCAACGACCACTTTGT |
|  | R | GCCAGACCCTGCACTTTTTAAG |
| SGLT2 | F | GGACTGTGCTAGCTGAAGGG |
|  | R | AACGCAGTTGGTCTTAGGCA |
| nephrin | F | CTGCCATCAGCAACTCTCCA |
|  | R | CTCTGCCTCTGTTGTGCTGA |
